# Vertical distribution of dioxins in soil of Bien Hoa airbase, Vietnam

**DOI:** 10.1186/s40064-015-1064-x

**Published:** 2015-06-30

**Authors:** Dang Thuong Huyen, Toshifumi Igarashi, Takuya Shiraiwa

**Affiliations:** Geo-Environment Department, Faculty of Geology and Petroleum Engineering, Ho Chi Minh City University of Technology, 168 Ly Thuong Kiet, Dist. 10, Ho Chi Minh City, Vietnam; Faculty of Engineering, Hokkaido University, Kita-ku, Sapporo City, 060-8628 Hokkaido Japan; Yagai-Kagaku Co., Ltd., Higashi-ku, Sapporo City, 065-0043 Hokkaido Japan

**Keywords:** Bien Hoa airbase, Dioxins, Soil, TEQ, Vertical distribution, Dominant isomer

## Abstract

Bien Hoa airbase is a known dioxin-contaminated hotspot in Vietnam. The contamination occurred during the Vietnam War at the site where dioxins were transported, stored, sprayed, and spilled in the area. Dioxins, which are cancer inducing substances, may transfer from the soil to food crops and finally to human beings living around the area. Many surveys of dioxins in soil, water, organisms, and human have been carried out in this study area since 2002. In this paper vertical distribution of dioxins in undisturbed soil cores were examined. Twelve soil samples from three drilled cores were collected to analyze dioxin levels according to the standard Japanese analytical method. The results showed that the toxicity equivalency quantity (TEQ) in one soil sample at a depth of 2.6 m reached 3,300 pg-TEQ/g-dw. High TEQs were also observed in the clay layer. This anomaly of dioxin concentrations could be attributed to the affinity of dioxins for the clay layer. The isomer patterns in the soils were different from those in the soil of Hokkaido in that 2,3,7,8-tetrachlorinated dibenzo-p-dioxin (TCDD) was the most dominant in the soil sample. This indicates that the dioxins originate from a defoliant Agent Orange disposed at the site after the Vietnam War.

## Background

Polychlorinated dibenzo-*p*-dioxins and polychlorinated dibenzofurans (PCDD/Fs) are known as hydrophobic organic compounds (HOCs) subject to long-range transport via vapour and particle-bound phases (Bergknut et al. 2010). The form of PCDD/Fs almost inexorably stabilized during combustion (Altarawned et al. 2009). These compounds are also formed by natural combustion processes, such as bushfires and volcanoes, as well as being unintentional byproducts of chemical reactions and incomplete combustion processes involving sources of chlorine and carbon (Rappe et al. 1987; Rappe 1996). They are harmful to humans when exposed mostly via the consumption of animal products (Elskens et al. 2013).

The source and distribution of PCDD/Fs were studied in Japan by Kakimoto et al. (2006), in Australia by Birch et al. (2007), and in a typical area of the studied district of eastern China by Liu and Liu (2009). In Huyen et al. (2013) has reported a much more comprehensive study associated with dioxin sources, environmental contamination status in Chinese environmental matrices on national scale. According to their studies, PCDD/Fs concentrations in the sediments of estuaries were higher (Birch et al. 2007). TEQ in soil and sediment samples decreased with an increase in the distance from the pollution sources (Liu and Liu 2009).

Vertical distribution of PCDD/Fs was reported by Czucwa et al. (1984) for a trend in sediment cores above the groundwater level of Isle Royale, Lake Superior. Götz et al. (2007), Bergknut et al. (2010), and Bulle et al. (2011) reported that PCDD/Fs concentrations decreased with depth in Germany, Sweden, and Canada, respectively. The concentrations of both organic matter and PCDD/Fs decreased with depth (Bergknut et al. 2010; Bulle et al. 2011). Kakimoto et al. (2006) showed that dioxins in soils were released with increased irrigation of water in the rice fields. In these soils, HOCs including PCDD/Fs were reported to increase with increasing amount of organic matter, and the concentrations of HOCs differed in the surface soils, deep soils and peat samples (Bergknut et al. 2010).

The survey of PCDD/Fs concentrations near the ground surface has been conducted in Bien Hoa airbase because this airbase was used to transport, store, spray, and spill dioxins during the Vietnam War (Office of the National Steering Committee 33, Monre and Hatfield Consultants 2011). In this report, the concentration of 2, 3, 7, 8-TCDD and TEQ in surface soils less than 10 cm deep were primarily measured, and only a few data of the concentrations in soils deeper than 20 cm were reported.

The reports mentioned above concern mainly on sources, horizontally spatial distribution in soil, and vertical distribution of dioxin in sediments. None of vertical distribution of dioxin is significantly considered in porous media. Therefore, the vertical distribution of PCDD/Fs concentrations has never been understood satisfactorily. In this study, the distribution was measured to characterize the mobility of PCDD/Fs by drilling three boreholes and taking undisturbed soil cores in the airbase.

## Study site and methods

### Study site

**Figure 1:**
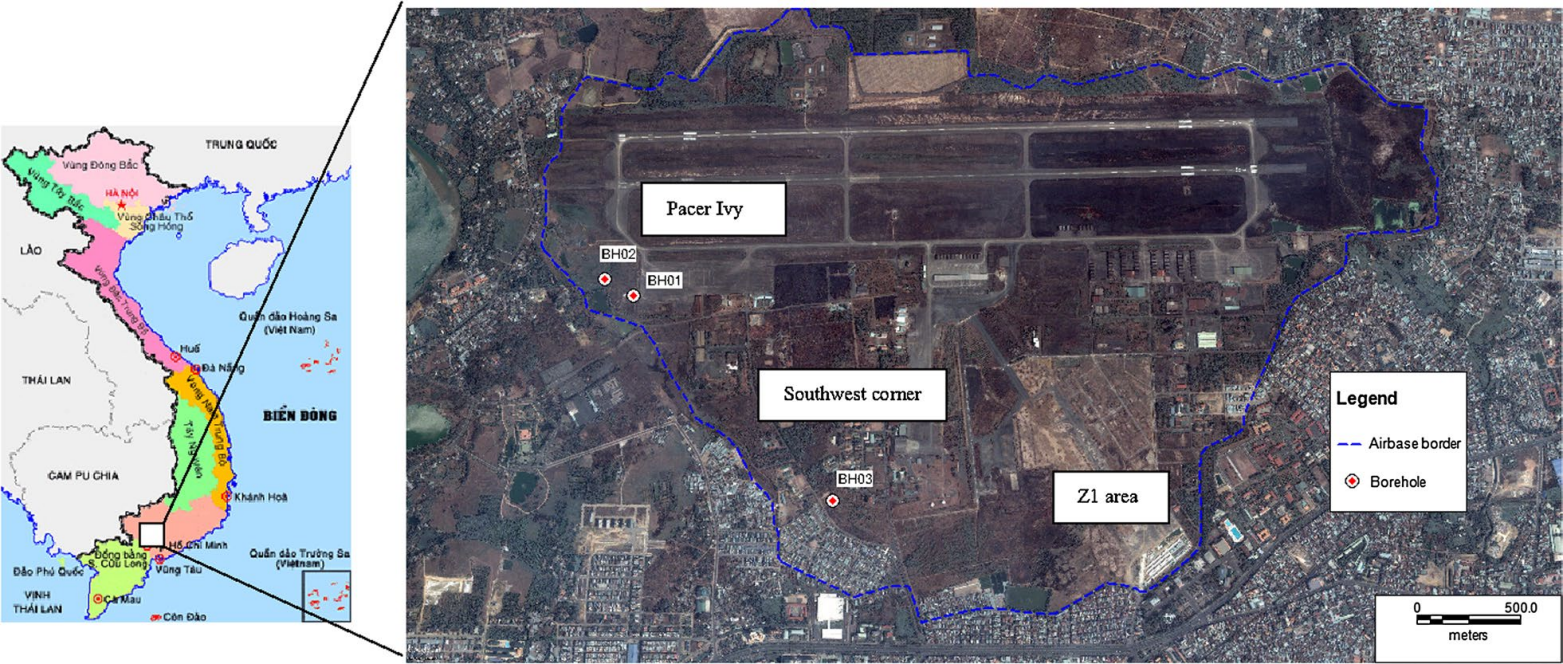
The Bien Hoa airbase (modified from Vietnam Embassy in Japan 2014; Google Map 2014).

The study area is located in Bien Hoa city of Dong Nai province (Figure 1). The distance between Bien Hoa airbase and Dong Nai River (the river supplies water not only for residents of Dong Nai province but also for those living in Ho Chi Minh City and other vicinities) is approximately 500 m. The airbase has a higher elevation than those of the surrounding areas, so contaminated groundwater flows from the airbase to the lower areas such as Bien Hung lake, Dong Nai river, and surrounding residential areas.

The airbase is one of the largest dioxin contaminated area in Vietnam. Sources of dioxins include Agent Orange, Agent White, and Agent Blue, all of which were transported and stored in this site during the Vietnam War. More than 22.67 million liters of Agent Orange, 9.36 million liters of Agent White, and 3.39 million liters of Agent Blue are believed to have been handled in this area (US DOD 2007; Young and Andrews 2007). Surveys of dioxins have been done since 2001 (Schecter et al. 2001, 2002; Dwernychuk et al. 2002; Dwernychuk 2005; Office of the National Steering Committee 33, Monre and Hatfield Consultants 2011), but these were only the shallow ground surface (<10 cm). Some soil samples in this shallow depth showed concentrations of dioxins several thousand higher than the Vietnamese standards. It was recommended that the contaminated soil should be treated immediately in the airbase (Vu-Anh et al. 2008; Office of the National Steering Committee 33, Monre and Hatfield Consultants 2011).

According to the information provided by the present department commander, and Office of the National Steering Committee 33, Monre and Hatfield Consultants (2011), Bien Hoa airbase has three dioxin hot spot zones. The first is Pacer Ivy with an area of ca. 20 ha and is still being surveyed. The highest concentration of TEQ measured at the surface soil was 28,600 pg-TEQ/g-dw. Pacer Ivy was used as a garrison and disposal site of the clothes of soldiers during the war. The second is the Southwest Corner (known as football stadium) with an area of 1.2 ha and is also being surveyed. This area was used as an infirmary for wounded soldiers, and the highest concentration of TEQ measured in the surface soil was 65,500 pg-TEQ/g-dw. The third is Z1 with an area of ca. 4.7 ha, which was used as an isolated landfill of 94,000 m^3^ of contaminated soil. The highest concentration of TEQ in the surface soil was 35,900 pg-TEQ/g-dw.

### Methods

#### Sampling

Three boreholes, BH01, BH02, and BH03, were drilled in the study site for collecting undisturbed soil samples. Two boreholes were dug in the Pacer Ivy area, while the third one was in the Southwest Corner of the airbase. Distances from BH01 to BH02, and from BH01 to BH03 are 170 and 1,360 m, respectively. The groundwater levels were shallow: GL-1.2 m at BH01, GL-1.1 m at BH02, and GL-5.1 m at BH03. All of the cores were transported to the Ho Chi Minh City University of Technology for analysis. Twelve undisturbed soil samples with approximately 5 cm in thickness were also collected based on the texture of soil. These samples were sealed with aluminum foil, and sent to Japan for analysis.

#### Chemical analysis

**Figure 2:**
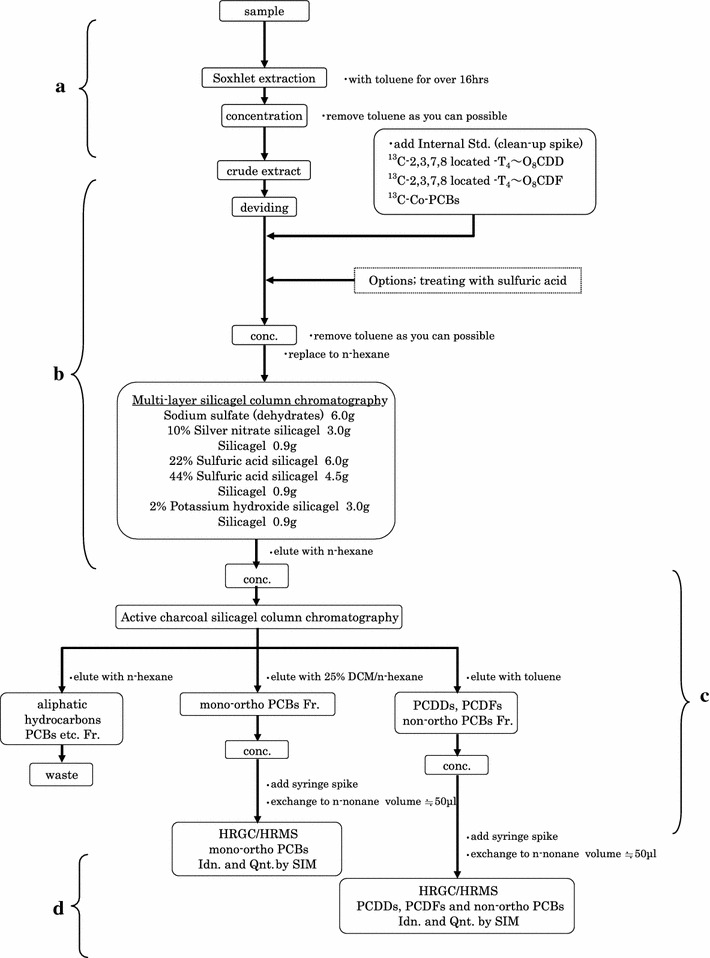
Analytical procedure flow chart.

Dioxin analysis in soil was carried out based on the standard analytical method in Japan (Ministry of Environment 2009) as shown in Figure 2. Soil samples were dried up under room temperature. Eight grams of each soil sample were placed in a thimble filter, and then, treated by Soxhlet extraction using toluene for more than 16 h (part a in Figure 2). The extracted crude solvent was evaporated, messed up to 100 ml, and divided by several aliquots (i.e., primarily by 0.1 ml and secondary by 90 ml). With adding internal standards as a clean-up spike in the separated solvent, the aliquot was evaporated, replaced to hexane, injected into a multi-layer column chromatograph with normal hexane (part b in Figure 2).

After the elution, effluent from the multi-layer column chromatograph was evaporated again, and the resulting product was injected into an active charcoal column chromatograph first with hexane, followed by 25% dichloromethane/hexane (for mono-ortho PCBs fraction), and then finally with toluene (for non-ortho PCBs fraction and PCDD/Fs). Each eluted fraction for analysis was purged by N_2_ gas to approximately 50μl, and taken in a vial bottle (part c in Figure 2). The sample was provided for a gas chromatograph–mass spectrometer (GC–MS, JEOL, Japan). WHO-TEF (2006) for TEQ calculation was adopted.

#### Quality assurance and quality control (QA/QC)

To enhance the quality of analyzed data, we checked a blank value regularly and analyzed the same sample three times for evaluating the variability. In addition, we calculated the recovery of samples within 50–120% according to the Japanese standard method.

## Results and discussion

### Vertical distribution of TEQ

**Figure 3:**
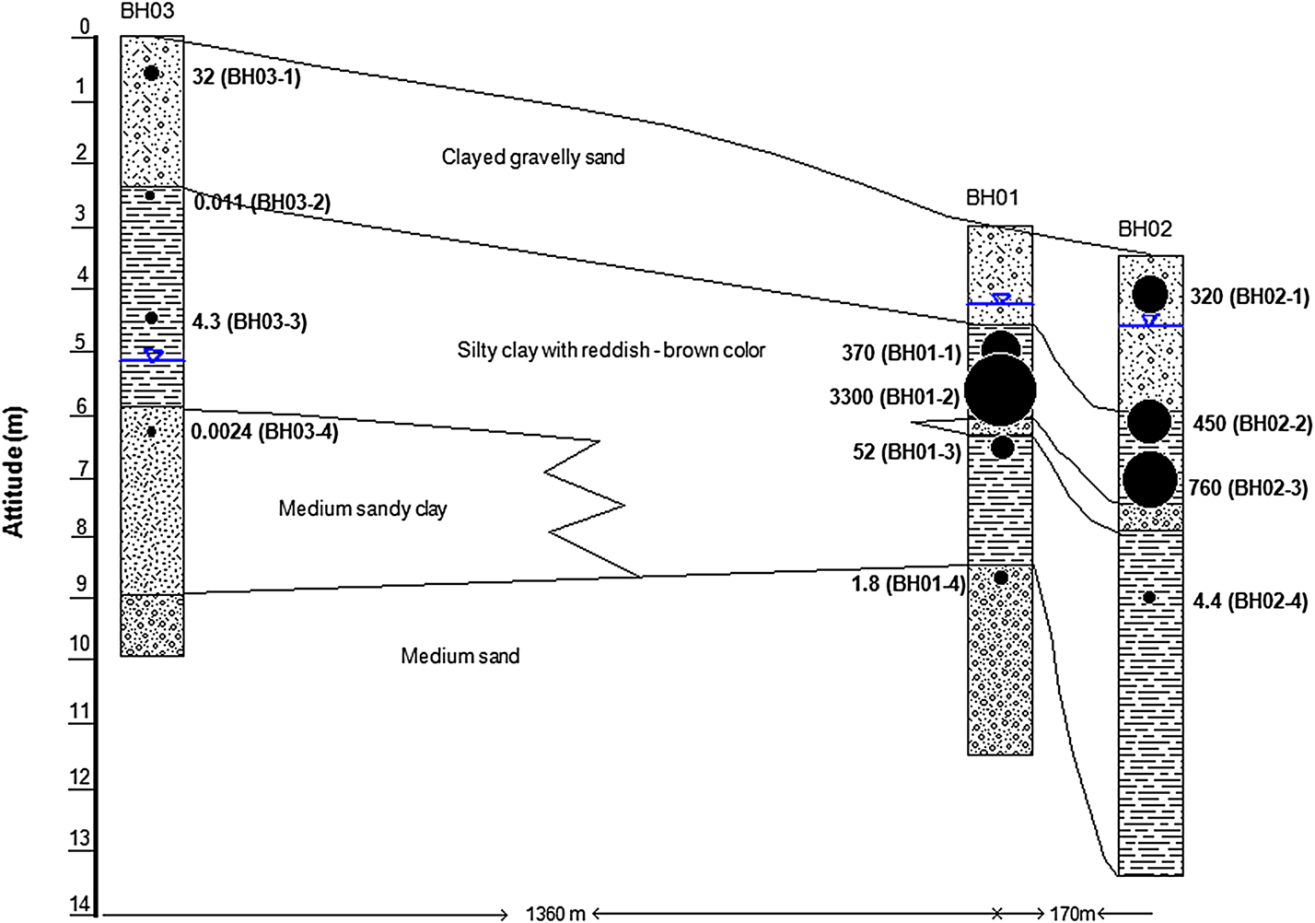
TEQ distribution in three boreholes. TEQ is shown using *black circles* (unit: pg-TEQ/g-dw). Soil texture and groundwater table in the boreholes are also shown in the figure.

The vertical distribution of TEQ is shown in Figure 3. The highest concentrations of 3,300 pg-TEQ/g-dw and 760 pg-TEQ/g-dw were observed at GL-2.5 m in BH01 and GL-3.5 m in BH02, respectively. Higher concentrations of dioxins were also found in the silty clay layer (BH01 and BH02). With depth, dioxin concentrations decreased, which could be attributed to the immobilization of dioxins in the impermeable layer. The upper clayey gravel-sand layer is likely to be used for backfilling materials for dioxin-bearing silty clay. In contrast, a much lower concentration of 32 pg-TEQ/g-dw was observed at the shallowest depth of GL-0.6 m in BH03, indicating that there was no significant source of dioxin near BH03. The above results suggest that the low-permeable silty clay layer prevents the migration of dioxins from the source layer to both the upper and lower layers for almost 40 years.

### Comparison of dioxin isomers at the study site with those at the other site

**Figure 4:**
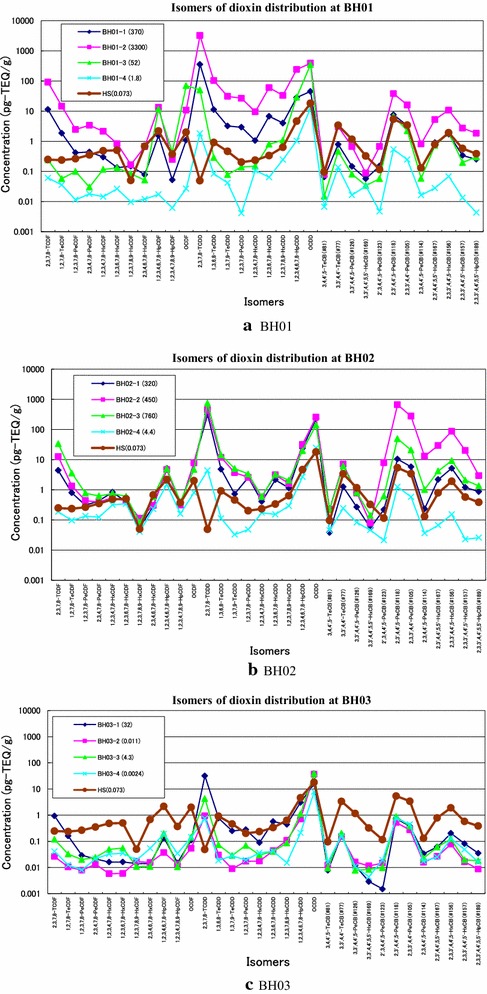
Isomer profiles of four collected soil samples and the other Japanese sample HS.

Figures 4a–c provide a comparison of isomers between the soil samples collected at the Bien Hoa airbase and that from Hokkaido, Japan (HS). The soil sample in Hokkaido is the typical uncontaminated one. The value of 0.3 pg-TEQ/g-dw as 2,3,7,8-TCDD corresponds to the detection limit of the analytical method used. The concentrations of 2,3,7,8-TCDD of soil cores from the three boreholes were much higher than that of the soil from Hokkaido, by comparing the concentrations of the other isomers. This indicates that the soil cores contain dioxins resulting from defoliant.

The concentrations of 2,3,7,8-TCDF and 1,2,7,8-TeCDF of soil cores were also higher than those of the soil of Hokkaido. TEQs are higher in all 12 soil samples when compared with the soil of Hokkaido (excluding of soil sample BH03-4). Isomer patterns of Co-PCBs of 12 soil samples and Japanese soil were similar. Sometimes, Co-PCB values of Hokkaido soil sample exceeded those of 12 soil samples. This may be due to the origin of dioxins.

**Figure 5:**
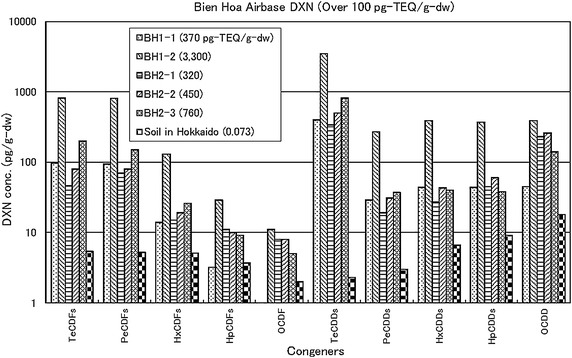
Comparison in congeners between soils of Bien Hoa and that in Hokkaido.

Congener profiles of dioxins between the soil samples with higher dioxin contents in Bien Hoa and the soil in Hokkaido were compared in Figure 5 to identify the source of dioxins indirectly, because it is difficult to obtain original defoliants used during the War. There was a dramatic difference in TeCDDs contents between the two sites. TeCDDs contents of Bien Hoa soils were higher than two orders of magnitude than that of Hokkaido soil whereas less than one- to two-orders magnitude was observed for the other congeners. This indicates that the source of TeCDDs in Bien Hoa soil results from defoliants used during the War.

### Contribution of 2,3,7,8 TCDD (or TCDD) to TEQ

**Figure 6:**
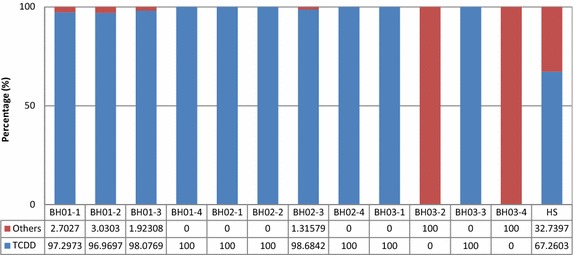
Percentage of TCDD to TEQ.

Figure 6 presents the percentages of 2,3,7,8-TCDD to TEQ. When the TEQ values were higher at BH01 and BH02, the percentages of 2,3,7,8-TCDD to TEQ approached 100%. However, when the TEQ values ranged from 0.0024 to 0.011 pg-TEQ/g-dw at BH03, the contribution of 2,3,7,8-TCDD to TEQ were ignored. This also means that the higher TEQ results from defoliants.

## Conclusion

Undisturbed soil samples were collected by drilling three boreholes in Bien Hoa airbase to analyze the vertical distribution of dioxins. High concentrations of dioxins were observed at GL-2.5 to -3.5 m in a silty clay layer of BH01 and BH02 boreholes. The distribution of the isomer profiles also showed that the higher concentrations of 2,3,7,8-TCDD was mostly caused by defoliants. In addition, the layer with higher concentration was restricted within a few meters. This means that although dioxins were relatively immobile in the subsurface environment consisting of low permeable layers, their migration should be evaluated and monitored in the long term.
